# Feasibility of applying a noninvasive method for sleep monitoring based on mouse behaviors

**DOI:** 10.1002/brb3.3311

**Published:** 2023-11-06

**Authors:** Ya‐Tao Wang, Yue‐Ming Zhang, Xu Wu, Chong‐Yang Ren, Zhe‐Zhe Zhang, Qi‐Gang Yang, Xue‐Yan Li, Gui‐Hai Chen

**Affiliations:** ^1^ Department of Neurology (Sleep Disorders) The Affiliated Chaohu Hospital of Anhui Medical University Hefei Anhui P. R. China; ^2^ School of Life Sciences University of Science and Technology of China Hefei P. R. China; ^3^ Departments of Anesthesiology, General Practice, or Critical Care The First Affiliated Hospital of Anhui Medical University Hefei Anhui P. R. China

**Keywords:** behavior, electroencephalogram, mice, sleep monitoring

## Abstract

**Introduction:**

Currently, electroencephalogram (EEG)/electromyogram (EMG) system is widely regarded as the “golden standard” for sleep monitoring. Imperfectly, its invasive monitoring may somehow interfere with the natural state of sleep. Up to now, noninvasive methods for sleep monitoring have developed, which could preserve the undisturbed and naïve sleep state of mice to the greatest extent, but the feasibility of their application under different conditions should be extensive validated.

**Methods:**

Based on existing research, we verified the feasibility of a sleep monitoring system based on mouse behaviors under different conditions. The experimental mice were exposed to various stresses and placed into a combined device comprising noninvasive sleep monitoring equipment and an EEG/EMG system, and the sleep status was recorded under different physiological, pharmacological, and pathophysiological conditions. The consistency of the parameters obtained from the different systems was calculated using the Bland–Altman statistical method.

**Results:**

The results demonstrated that the physiological sleep times determined by noninvasive sleep monitoring system were highly consistent with those obtained from the EEG/EMG system, and the coefficients were 94.4% and 95.1% in C57BL/6J and CD‐1 mice, respectively. The noninvasive sleep monitoring system exhibited high sensitivity under the sleep‐promoting effect of diazepam and caffeine‐induced wakefulness, which was indicated by its ability to detect the effect of dosage on sleep times, and accurate determination of the sleep/wakeful status of mice under different pathophysiological conditions. After combining the data obtained from all the mice, the coefficient between the sleep times detected by behavior‐based sleep monitoring system and those obtained from the EEG/EMG equipment was determined to .94.

**Conclusion:**

The results suggested that behavior‐based sleep monitoring system could accurately evaluate the sleep/wakeful states of mice under different conditions.

## Graphical abstract

The noninvasive sleep monitoring system was synchronized with the standard electroencephalogram to record sleep time. The results of consistency test proved the feasibility of the noninvasive sleep monitoring system in recording sleep time in different states.

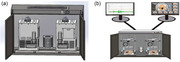

## 1 INTRODUCTION

Insomnia is defined by the self‐reported presence of difficulty with sleep, and its emergence is often associated with social, environmental, and psychological stresses (Wang et al., 2020). The prevalence of insomnia varies in different countries, ranging from 10% to 40% among young and middle‐aged people (Kische et al., 2016); therefore, the etiology and treatment of insomnia remains a research priority. The presently available clinical treatments for chronic insomnia are primarily symptomatic and have poor efficacy. The lack of understanding of the neurobiological mechanism of chronic insomnia is attributed to the fact that a mammalian model of insomnia has not been developed to date. Rodents, including mice and rats, are commonly used in sleep research owing to similarities with human beings in terms of homeostatic regulation of sleep, circadian rhythm, and sensitivity to internal/external stimuli (Drinkenburg et al., 2015; Hagenauer & Lee, 2013; Panagiotou et al., 2021). To date, the most prevalent approach for sleep monitoring in rodents involves electroencephalogram (EEG)/electromyogram (EMG) recording, which is described as the “golden standard” in sleep monitoring studies. The basic principle of EEG/EMG recording involves the determination of the stage of sleep based on the frequency of particular EEG and EMG waves in the sleep state (Ancoli‐Israel et al., 2003). In this method, the rodents are anesthetized prior to assessment, and holes are drilled in their skulls, following which electrodes are installed and the rodents are allowed to recover for a week. This strategy allows professional technicians to analyze the sleep/wake times, depth of sleep, and other indicators with high accuracy under a relatively restrained state. However, the EEG/EMG recording approach is an invasive method and has several disadvantages, which are described hereafter. First, the surgical procedure necessary for implanting the electrodes could itself be a stressor which might disrupt the normal sleep state to a certain extent. Second, tracking sleep parameters at different stages during the lifetime of a rodent would aid in understanding the neurobiological mechanisms of age‐related sleep disorders (Mander et al., 2017); however, the long‐term and repeated evaluation of EEG/EMG recordings during the lifetime of a single animal is hardly feasible. Lastly, certain pathological rodent models, including rodent models of sepsis, might be unable to withstand the surgical treatment necessary prior to EEG/EMG recordings (Stortz et al., 2019). The development of a convenient and noninvasive strategy for monitoring sleep throughout the lifetime of an individual rodent is extremely necessary for meeting the diverse requirements of research studies and different ranges of application.

To date, studies have aimed to develop few strategies for replacing the EEG/EMG recording system and achieved some improvements. For instance, Mang et al. (2014) used a piezoelectric device for determining the sleep/wake states of CFW Swiss Webster mice and monitoring the breathing rhythm during sleep, and the device was reported to have an accuracy of 90%. The piezoelectric system developed by Mang et al. (2014) is the only commercially available equipment for noninvasive sleep monitoring and has been used to detect the effects of first‐line clinical drugs, including verapamil and moricizine, on sleep (Burish et al., 2021; Han et al., 2021). Bastianini et al. employed whole‐body plethysmography (WBP) for detecting the subtle changes in pressure in the body cavity during sleep/wake states for assessing sleep times and breathing rhythms. The coefficient between the sleep data obtained using this equipment and those obtained from EEG/EMG recordings was over 0.9 (Bastianini et al., 2017). The sealed WBP chamber has an internal volume of only 1 L and restricts the free space for roaming; the apparatus is therefore commonly used in studies investigating lung function in mice (Chu et al., 2019; Vaickus et al., 2010). Similarly, Piilgaard et al. (2023) demonstrated that home‐cage activity tracking could be used for narcolepsy phenotyping, which provided effective measure to facilitate faster and larger scale assessments of narcolepsy disease. Additionally, Pack et al. demonstrated that the sleep time was related to the duration for which the mice maintained a motionless posture, and this duration was determined to be over 40 s in mice. The study compared video‐defined sleep with EEG‐defined sleep under normal physiological conditions in C57BL/6J mice and determined that the average correlation between the sleep data obtained from video recording and EEG/EMG recording was 92% (Pack et al., 2007). In another study, Fisher et al. (2012) verified that the sleep times in the recorded videos were consistent with those determined from EEG recordings in C57BL/6J mice under different pharmacological states induced by caffeine and zolpidem. The behavior‐based sleep monitoring system is extremely convenient and can preserve the natural sleep state of mice to a considerable extent. However, the suitability of behavior‐based sleep monitoring system for more application scenarios, including certain pathophysiological states, such as sepsis or depression‐like behaviors induced by chronic social defeat stress (CSDS), as well as in motor retardation accompanied by prolonged immobility (Zheng et al., 2016). Furthermore, it is necessary to determine whether the 40 s rule determined by Pack et al. is applicable to other strains of mice.

Currently, there is not a mouse model of chronic insomnia that is widely acknowledged. Seugnet et al. (2009) obtained a heritable and stable phenotype of flies with chronic insomnia by using a behavior‐based sleep monitoring system, which provided inspiration for the development of a method for long‐term and repeated sleep monitoring. It seems to be a good approach to obtain mice with a stable short sleep phenotype by screening short sleep mice for multiple passages. We therefore developed the corresponding software and hardware system based on the study by Pack et al., which reported that the mice that remain motionless for over 40 s can be regarded as sleeping. By comparison with standard EEG/EMG equipment, we first repeatedly determined the accuracy of behavior‐based sleep monitoring system under physiological and pharmacological conditions. Moreover, we expend the results into other strains. Next, we examined relatively extreme cases, including stress caused by cat litter exposure and depression induced by lipopolysaccharide (LPS)‐treatment or CSDS, for extensive validation. The results suggested that behavior‐based sleep monitoring system can be feasibly applied for sleep monitoring under different conditions. The study provides a novel perspective for the development of noninvasive long‐term and repeated sleep monitoring.

## 2 MATERIALS AND METHODS

### 2.1 Animals

CD‐1 and C57BL/6J male mice, aged 6–8 weeks old, were purchased from Zhejiang Vital River Laboratory Animal Device Co., Ltd. (NO. 20210908Abzz0619000360). The animals were maintained at a constant temperature of 22–25°C and a relative humidity of 55% ± 5%, under a 12/12 h light/dark cycle in which the lights were turned on at 08:00 h. The mice were allowed ad libitum access to food and water. The mice were operated at around 3 months of age, and their sleep behaviors were subsequently recorded. Following completion of the surgery required for EEG/EMG, the mice were immediately divided into separate groups for subsequent analysis, with six mice in each group. Following the completion of experiments, mice were anaesthetized with pentobarbital sodium (concentration: 2%; dosage: 0.1 mL/20 g) followed by cervical dislocation. All the experimental procedures were approved by the Experimental Animal Committee of Anhui Medical University (approval number: LLSC20190710).

### 2.2 Surgery for the installation of EEG/EMG electrodes

The mice were anesthetized with a cocktail of ketamine (100 mg/kg) and xylazine (25 mg/kg) and fixed on a stereotaxic frame. A midline incision was made in the scalp of the upper part of the skull, and the skull was subsequently exposed. In order to record the EEG waves from the frontal–parietal lobes, four holes were drilled through the skull without breaking the dura mater. For optimal EEG alignment, the front edge of the implant should be placed 3–3.5 mm anteriorly to the bregma, and all the four screws need to be kept in this configuration in the cerebral cortex of a fully grown mouse. In this study, four tiny stainless steel screws were placed in the four holes for fastening the headmount. Two standard stainless steel EMG electrodes were inserted into the dorsal nuchal muscle for recording the muscular activity. Dental cement was used to fix the electrodes onto the skull and cover the screws, following which the wound was sutured and antibiotics were smeared around the wound. The next phase of monitoring was performed after a week of recovery. The EEG signals were subsequently sampled at 400 Hz, and high‐pass wavelength filters of 25 and 100 Hz were used for EEG and EMG, respectively.

### 2.3 Equipment for synchronous video and EEG recordings

**FIGURE 1:**
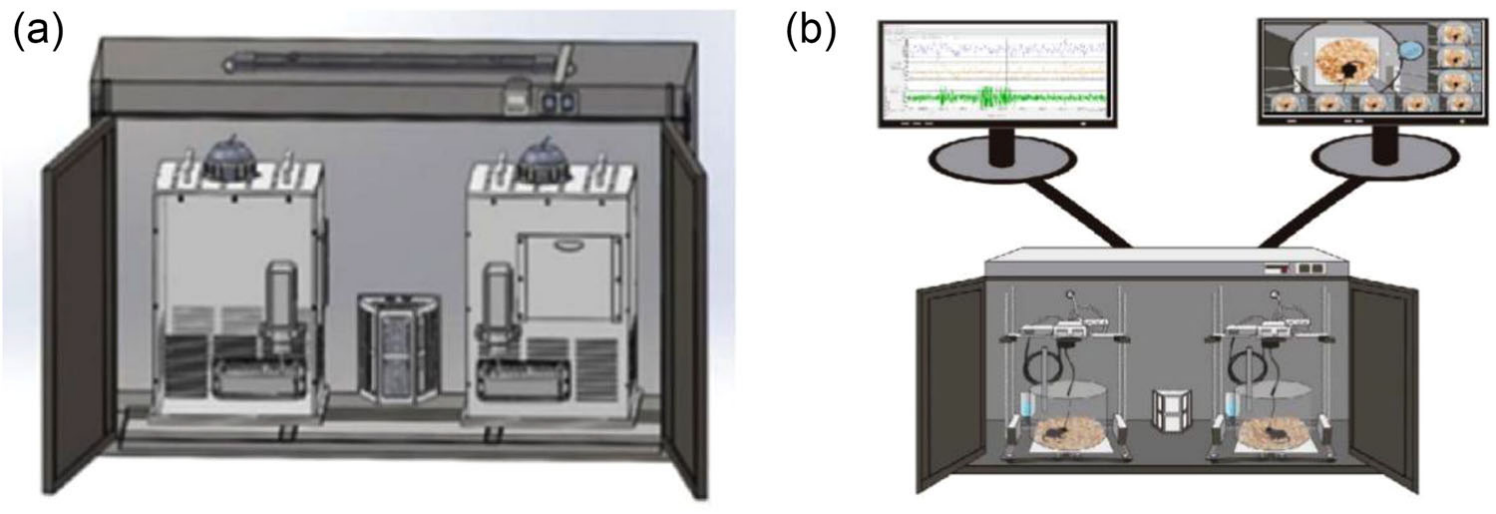
Schematic depicting the sleep monitoring equipment (a) schematic depicting the noninvasive video monitoring equipment. (b) The infrared camera of the video monitoring system was placed above the cage to ensure that the camera is able to monitor the activities of the mice. The sleep data can be simultaneously recorded by two different systems.

A recording cabinet was constructed in this study, which was equipped with a ventilation system and maintained under a light/dark cycle of 12/12 h, in which the lights were turned on at 08:00 h and switched off at 20:00 h. The equipment could be sheltered from external light and potential signal interference (Figure 1a). The camera of the noninvasive monitoring device was combined with the EEG/EMG recording system for simultaneous recording (Figure 1b). The real‐time behaviors of the mice were recorded using a camera with infrared night vision (Dahua, version: 1230C‐A, resolution ratio: 1920 × 1080p, video file format: AVI) installed 40 cm above the bottom of the cage. In cages with identical design parameters, a high‐definition camera was utilized to generate a real‐time video stream. The video frame was adjusted to ensure that the active area of the mice remained intact and no moving objects outside the cage interfered with the video. The Vibe algorithm was employed to detect the number of pixels where the target (mouse) moved in the camera lens frame. The threshold of mouse activity was set based on the number of pixels detected. During the preexperimental phase, we tested the threshold of mouse activity. To ensure the accuracy of detecting mouse movement, the algorithm provides relevant parameters for the host computer software debugging. In the preexperimental phase, we executed the test using a 5% numerical gradient from 0% to 100%. The fluctuations in abdominal breathing and ear movements were regarded as usual activities during sleep, and their values were set below the threshold as they would not trigger arousal. By comparing the consistency with EEG/EMG sensors, the suitable motor threshold was identified. Finally, it is employed in formal experiments. The value of the video‐defined sleep parameter in our experiment was set at 40 s (Table 1). The mice were placed in the cage and allow to adapt prior to formal monitoring. Following acclimatization, the sleep data were simultaneously recorded by the EEG/EMG system and our video capturing device. After monitoring, the video‐based inactivity times of the mice in each of the cages were determined by its software. The sleep times recorded by the EEG/EMG system were analyzed using the Sirenia Sleep software (Pinnacle Device Inc.), following which experienced sleep scorers used a 10 s epoch for manually scoring the sleep/wake times in the target time. The total sleep time consisted of the duration of sleep during the rapid eye movement (REM) and non‐REM (NREM) periods.

**TABLE 1:** Consistency of different parameters.

Mouse strain	Parameter	35 s	40 s	45 s
C57BL/6J	Limits of agreement(s)	−652.8 to 384.4	−226.2 to 448.3	−326.3 to 609.2
	Consistency (%)	93.1	94.4	94.0
	Total sleep time from videos	48,945	44,728	42,792
	Total sleep time from EEG	45,673	47,393	46,187
CD‐1	Limits of agreement(s)	−751.5 to 482.1	−405.9 to 595.6	−237.5 to 792.1
	Consistency (%)	93.1	95.1	94.4
	Total sleep time from video	47,948	42,668	38,874
	Total sleep time from EEG	44,715	44,995	45,530

### 2.4 Intraperitoneal injection of caffeine, diazepam, and saline

In order to investigate the effects of caffeine on sleep times, three groups of mice were separately treated with different doses of caffeine (5, 10, and 15 mg/kg) (Pack et al., 2007) by intraperitoneal injection at 08:00 h, whereas the control group was intraperitoneally injected with saline at the same time. The two diazepam‐treated groups were intraperitoneally injected with 2.5 or 5 mg/kg diazepam at 21:00 h, whereas the mice in the control group were intraperitoneally injected with saline at the same time. The injection was completed within 5 min, and the C57BL/6J mice were immediately placed in the cages following injection and the recording was initiated.

### 2.5 Cat litter stress

In order to analyze the sleep data under stressful conditions, dirty cat litter was placed in the cages of C57BL/6J mice at 08:00 h, and fresh, unused cat litter was placed in the cages of the mice in the control group.

### 2.6 Depression models

The depression models used in this study included CD‐1 mice subjected to inflammation stress and C57BL/6J mice exposed to CSDS. The CD‐1 mouse model of inflammation was generated by intraperitoneal injection of 0.83 mg/kg LPS for inducing inflammation (Jiang, Liu, et al., 2017), whereas the control group was intraperitoneally injected with saline at the same time. Sleep time analysis and behavioral tests were performed on the second day after injection. The C57BL/6J mouse model of CSDS was generated by placing C57BL/6J mice in a cage containing selected aggressive CD‐1 mice, and the invading C57BL/6J mice were challenged by the CD‐1 mice within 10 min. After 10 min of social defeat, the C57BL/6J mice were separated from the aggressive CD‐1 mice with a perforated plexiglass. The following day, these C57BL/6J mice were placed in another cage containing different aggressive CD‐1 mice for preventing habituation. The experiment lasted for 10 days, and social defeat occurred at the same time every day (Jiang, Wang, et al., 2017). The control group was placed in an identical cage and was separated from the aggressive CD‐1 mice by a perforated plexiglass for allowing sensory contact for 24 h. The CD‐1 mice were altered on a daily basis for preventing habituation in the control C57BL/6J mice. Following the completion of these procedures, one half of the mice in each group (*n* = 6) were placed into monitoring cages for adaptation, following which the sleep time was simultaneously recorded on the second day. The other half of the mice in the different groups (*n* = 6) were tested for depressive‐like behaviors. The behavioral tests included the tail suspension test (TST) and the forced swimming test (FST). In the TST, the tail was affixed with a tape, and the mouse was suspended in an upside‐down position. The test lasted for 6 min and the total duration for which the mouse remained immobile in the last 4 min of the test was determined as the immobility time. In the FST, a transparent bucket with a diameter of 10 cm and a height of 25 cm was filled with warm water (temperature of 23–25°C) at a depth of approximately 10 cm. The test lasted for 6 min and the total duration for which the mouse remained immobile within the last 4 min was recorded as the immobility time.

### 2.7 Statistical analyses

The normally distributed data are presented as the mean ± standard error of the mean. The sleep/wake times within 24 h were recorded simultaneously under different conditions and divided into 24 × 1 h segments. The consistency was calculated by using the Bland–Altman method comparison in the Column analyses section of the GraphPad Prism 8 software (Bland & Altman, 1986). Repeated measures analysis of variance (ANOVA) was used to compare the effect of the different doses of caffeine and diazepam. Student's *t*‐test was used to analyze the effect of pharmacological (caffeine and diazepam) and pathophysiological (predator odor) stresses and depression‐associated behaviors. The differences were regarded significant at *p* < .05. All the statistical analyses were performed with GraphPad Prism, version 8.0.

## 3 RESULTS

### 3.1 Consistency of different parameters in control C57BL/6J and CD‐1 mice

**FIGURE 2:**
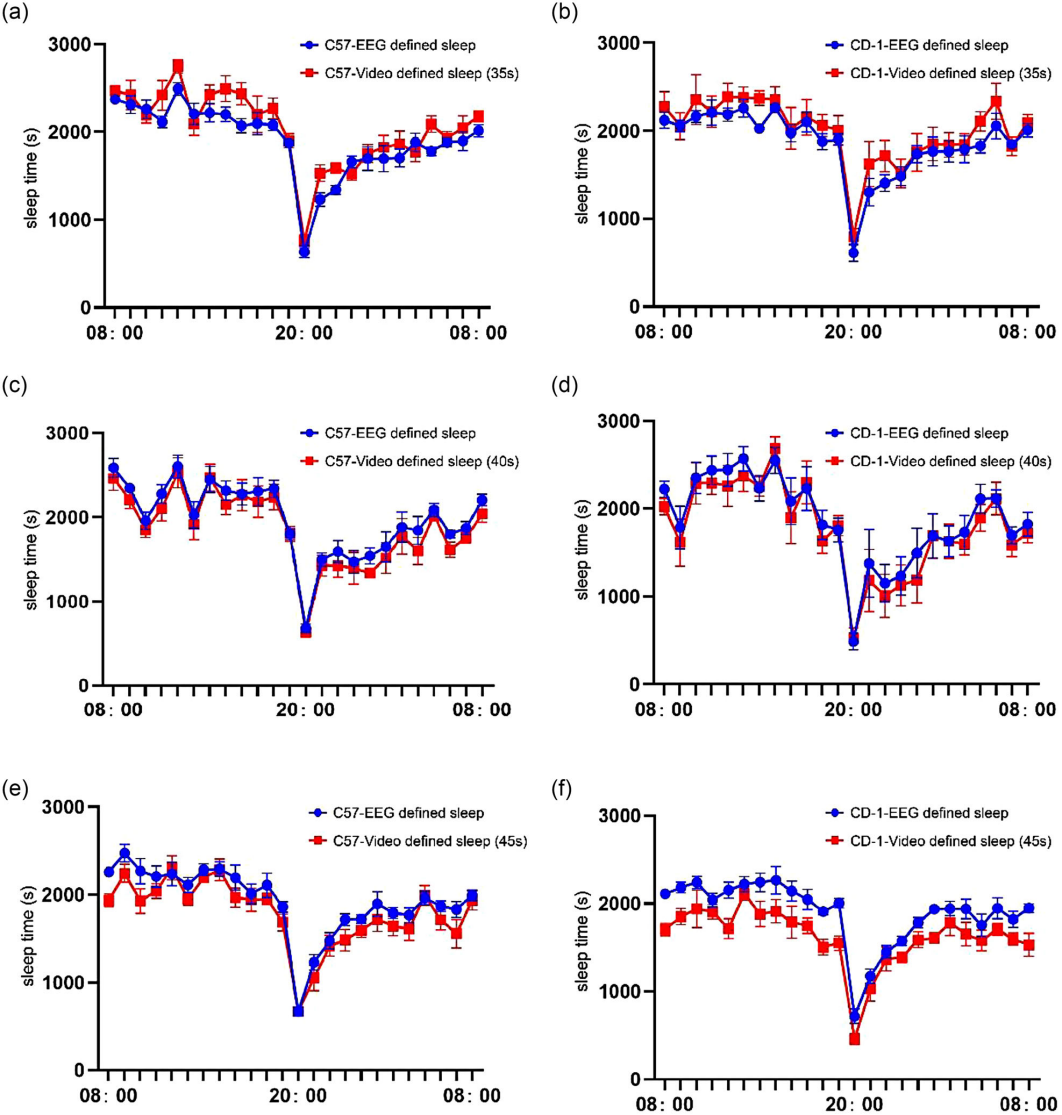
Time course of the changes in electroencephalogram (EEG)/electromyogram (EMG)‐defined sleep and video‐defined sleep for the three parameters of healthy C57BL/6J and CD‐1 control mice. (a and b) Time course of changes detected by video sleep monitoring system and EEG/EMG system of C57BL/6J and CD‐1 mice in 35 s group. (c and d) Time course of changes detected by video sleep monitoring system and EEG/EMG system of C57BL/6J and CD‐1 mice in 40 s group. (e and f) Time course of changes detected by video sleep monitoring system and EEG/EMG system of C57BL/6J and CD‐1 mice in 45 s group.

Previous studies have demonstrated that an immobility time of 40 s is an effective metric for determining sleep/wake states (Fisher et al., 2012; Pack et al., 2007). It was therefore necessary to determine whether this parameter was applicable to the equipment developed in this study. We therefore selected three values of this variable for defining sleep, including inactivity for 35, 40, and 45 s, in the control C57BL/6J and CD‐1 mice (*n* = 6). As depicted in Table 1 and Figure 2a–f, we observed that the video‐based inactivity times detected by noninvasive sleep monitoring system was lower than that recorded by the EEG/EMG system for the 45 s group, whereas the video‐based inactivity times determined by noninvasive sleep monitoring system was higher than that recorded by the EEG/EMG system for the 35 s group. The coefficient between the sleep times recorded by noninvasive sleep monitoring system and those determined using the EEG/EMG system for the C57BL/6J and CD‐1 control mice in the 40 s group was highest among the three groups. Combining the results of the limits of agreement, consistency data, and total sleep time, we tentatively believed that 40 s was the appropriate parameter of the three to define immobility time as sleep. Moreover, more parameters are needed subsequently to verify the reliability of 40 s. Based on this value, we obtained the sleep data under different physiological conditions. The average sleep time per hour determined by behavior‐based system was 1863.6 ± 44.9 and 1777.8 ± 56.6 s in the control C57BL/6J and CD‐1 groups, respectively, and 1974.7 ± 42.3 and 1874.8 ± 55.0 s, respectively, as determined by the EEG/EMG equipment. The coefficient between the sleep times recorded by behavior‐based system and those obtained using the EEG/EMG equipment was 94.4% and 95.1% in the control C57BL/6J and CD‐1 mice, respectively. Based on the differences in sleep time recorded by our device and the EEG/EMG equipment within 24 h, the Bland–Altman 95% limits of agreement in the control C57BL/6J and CD‐1 mice were determined to be −226.2 to 448.3 and −405.9 to 595.6 s, respectively.

### 3.2 Sleep data of the C57BL/6J caffeine‐treated group

**FIGURE 3:**
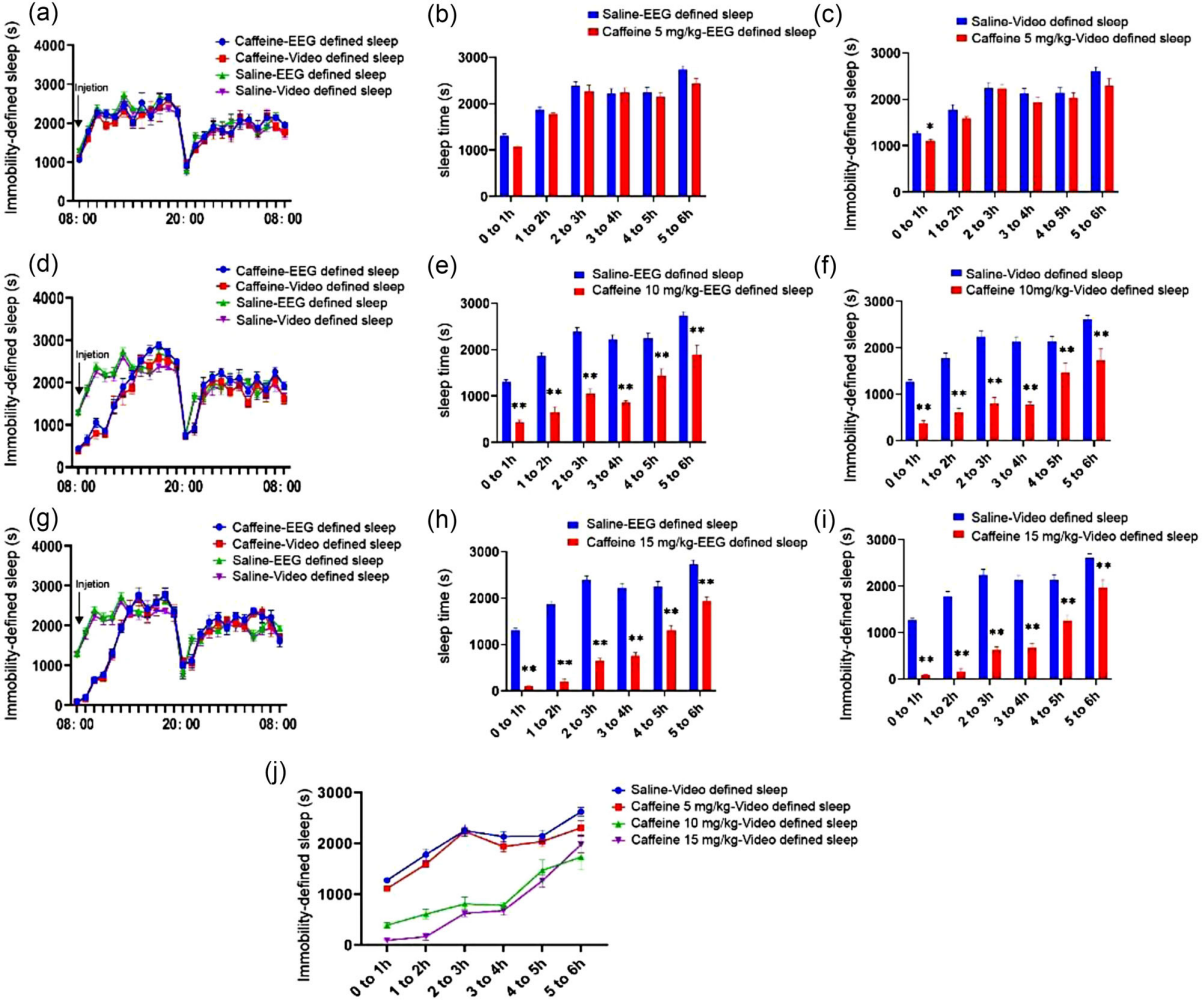
Effects of different doses of caffeine on the sleep parameters (a) time course of the changes in video‐defined sleep and electroencephalogram (EEG)/electromyogram (EMG)‐defined sleep in the groups treated with 5 mg/kg and saline. (b and c) The sleep time recorded by EEG/EMG device and video‐defined inactivity times of the group treated with 5 mg/kg caffeine and the saline‐treated group. (d) Time course of the changes in video‐defined sleep and EEG/EMG‐defined sleep in the group treated with 10 mg/kg and the saline‐treated group. (e and f) The sleep time recorded by EEG/EMG device and video‐defined inactivity times of the group treated with 10 mg/kg caffeine was significantly lower than that of the saline‐treated group within 6 h of intraperitoneal injection: (g) Time course of the changes in video‐defined sleep and EEG/EMG‐defined sleep in the groups treated with 15 mg/kg caffeine and saline. (h and i) The sleep time recorded by EEG/EMG device and video‐defined inactivity times of the group treated with 10 mg/kg caffeine was significantly lower than that of the saline‐treated group within 6 h of intraperitoneal injection. (j) Within 6 h of intraperitoneal injection, the results of sleep time or inactivity times in the three doses of caffeine group and saline group. *p < .05, **p < .01, saline versus caffeine.

In order to further determine the applicability of behavior‐based system under a hyperarousal state, the mice were injected with different doses of caffeine and the sleep times in the groups treated with 10 mg/kg (Figure 3d–f) and 15 mg/kg (Figure 3g–i) caffeine for 6 h was lower than those of the saline‐treated group. However, the differences in sleep time between the group treated with 5 mg/kg caffeine, and the group injected with normal saline was only evident in the first hour following injection (Figure 3a–c). The results of repeated‐measures ANOVA revealed that the sleep time was reduced following treatment with caffeine (*F*_(3, 20)_ = 241.1, *p* < .01), and a high dose of caffeine (15 mg/kg) had a stronger effect on reducing the sleep time compared to the medium (10 mg/kg) and low (5 mg/kg) doses (Figure 3j).

### 3.3 Sleep data of the C57BL/6J diazepam‐treated group

**FIGURE 4:**
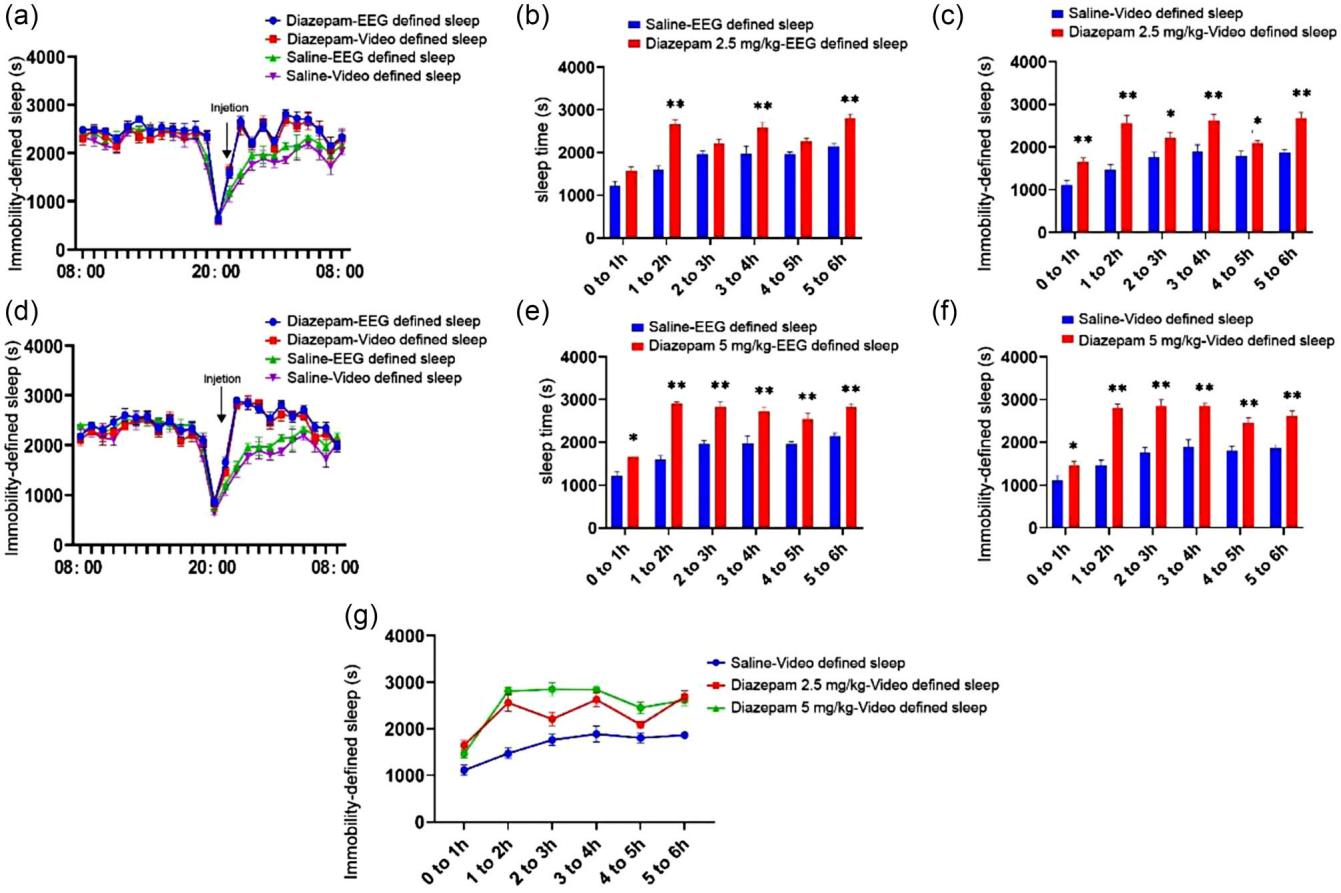
Effects of different doses of diazepam on the sleep parameters (a) time course of the changes in video‐defined sleep and electroencephalogram (EEG)/electromyogram (EMG)‐defined sleep in the groups treated with 2.5 mg/kg diazepam and saline. (b and c) The sleep time recorded by EEG/EMG device and video‐defined inactivity times of the group treated with 2.5 mg/kg diazepam was significantly longer than that of the saline‐treated group within 6 h of intraperitoneal injection. (d) Time course of the changes in video‐defined sleep and EEG/EMG‐defined sleep in the groups treated with 5 mg/kg diazepam and saline. (e and f) The sleep time recorded by EEG/EMG device and video‐defined inactivity times of the group treated with 5 mg/kg diazepam was significantly longer than that of the saline‐treated group within 6 h of intraperitoneal injection. (g) Within 6 h of intraperitoneal injection, the results of sleep times or inactivity times showed the sleep‐promoting effect of 2.5 and 5 mg/kg diazepam. *p < .05, **p < .01, saline versus diazepam.

**FIGURE 5:**
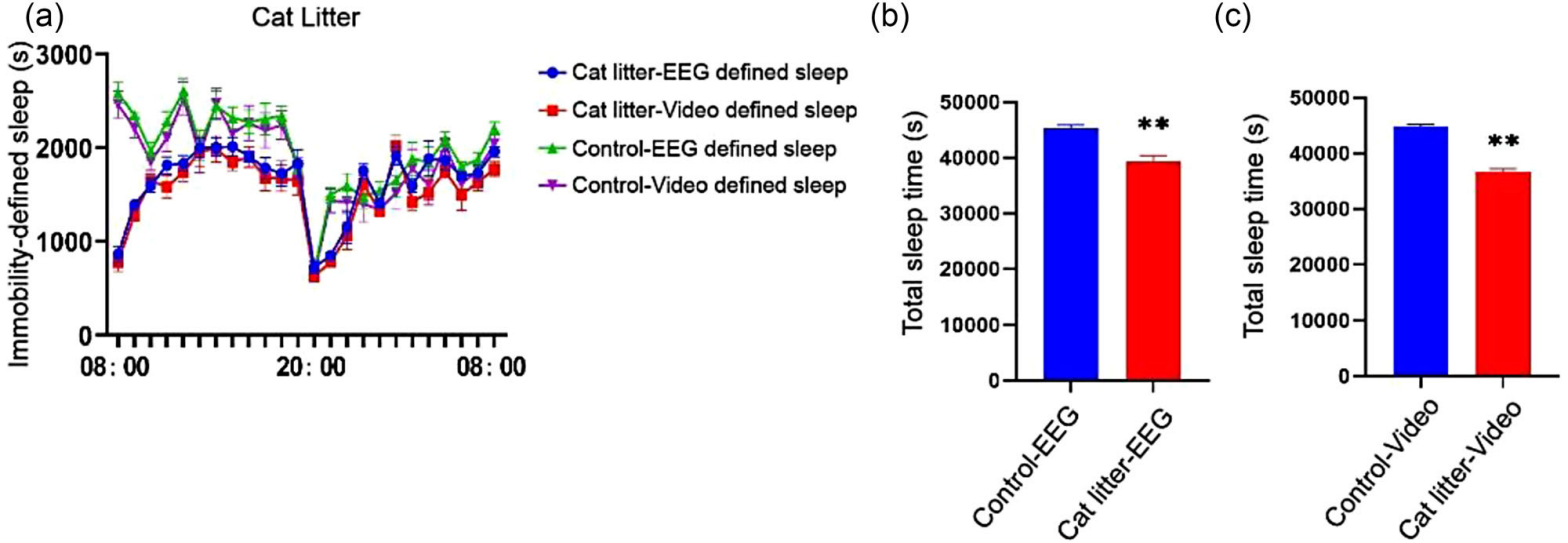
Effects of predator stress on sleep parameters (a) time course of the changes in video‐defined sleep and electroencephalogram (EEG)/electromyogram (EMG)‐defined sleep in the group exposed to cat litter. (b and c) Total sleep time of the group exposed to cat litter and the control group within 24 h. **p < .01.

The sensitivity of the video monitoring system was determined by intraperitoneal injections of different doses of diazepam. The sleep times of the mice treated with 2.5 mg/kg (Figure 4a–c) and 5 mg/kg (Figure 4d–f) diazepam were higher than those of the saline‐treated group. The results of repeated‐measures ANOVA revealed that the sleep time increased following diazepam treatment (*F*_(2, 15)_ = 57.95, *p* < .01). The sleep‐promoting effect of diazepam at a relatively high dose (5 mg/kg) was comparable to that at a relatively low dose (2.5 mg/kg) (Figure 4g). The results showed that behavior‐based system could detect the prolongation of video‐based immobility time due to diazepam injection.

### 3.4 Sleep data of C57BL/6J group exposed to cat litter

The total sleep time of the mice was reduced in comparison to that of the control group following exposure to predator odor (*p* < .01). The time course of the sleep time determined by behavior‐based system was highly consistent with that obtained using the EEG/EMG system under conditions of stress (Figure 5a–c).

### 3.5 Results of behavior tests and total sleep time analysis in depression groups

**FIGURE 6:**
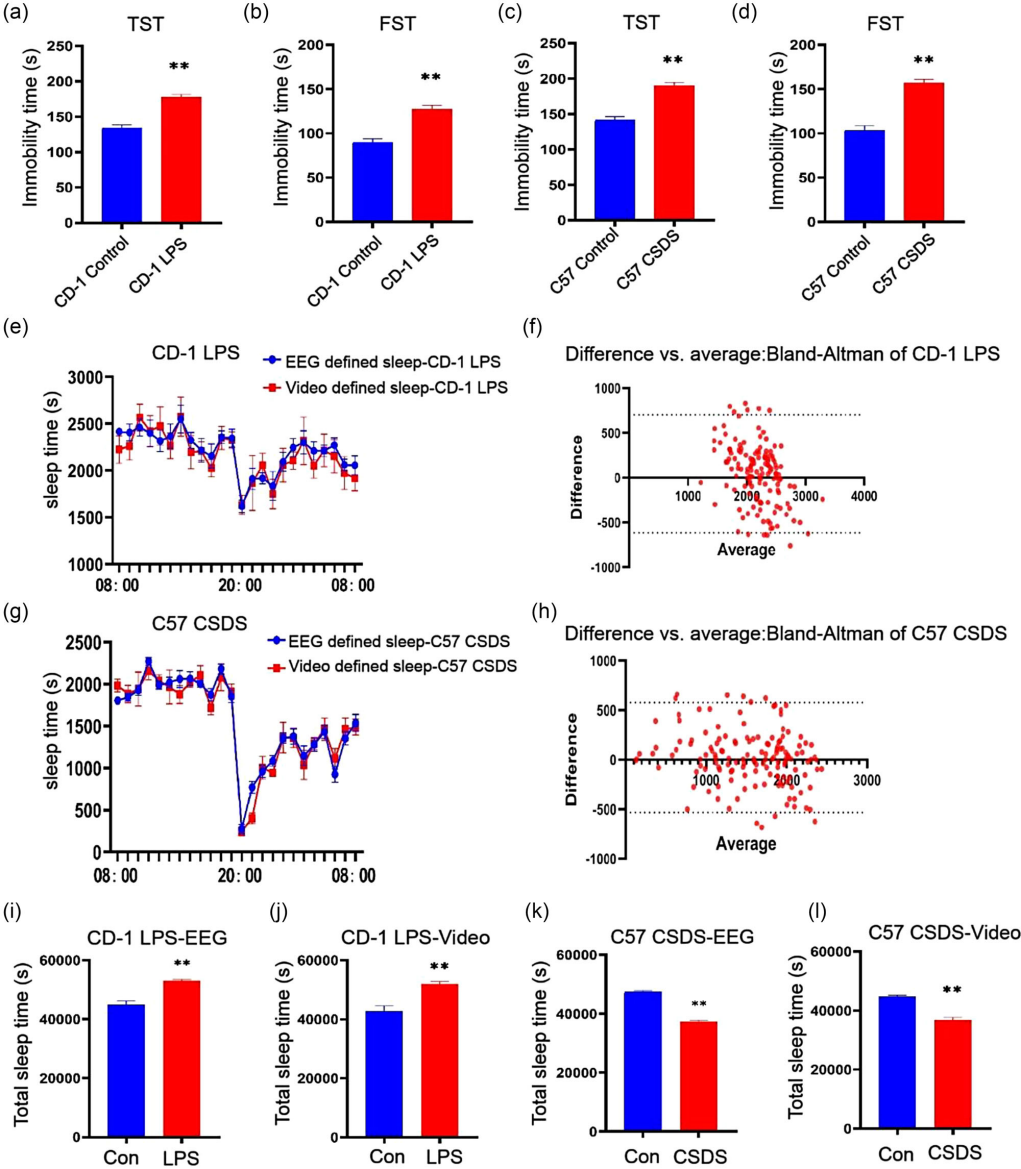
Changes in behavior and sleep in the two murine models of depression (a–d) immobility time of the two mouse models of depression in tail suspension test (TST) and forced swimming test (FST). (e) Time course of the changes in video‐defined sleep and electroencephalogram (EEG)/electromyogram (EMG)‐defined sleep in CD‐1 mice. (f) The Bland–Altman method was used to evaluate data consistency in the CD‐1 group. (g) Time course of the changes in video‐defined sleep and EEG/EMG‐defined sleep in C57BL/6J mice. (h) The Bland–Altman method was used to evaluate data consistency in the C57BL/6J group. (i–l) Total sleep time of the mouse models of depression and the control groups. **p < .01.

In order to further explore whether behavior‐based system can be applied to murine models of depression, an LPS‐treated group (*n* = 6) and a CSDS group (*n* = 6) were generated in this study. The total immobility time of the control and LPS‐treated CD‐1 mice in the TST was determined to be 133.7 ± 4.6 and 177.6 ± 4.0 s, respectively (Figure 6a), and the total immobility time of the control and LPS‐treated CD‐1 mice in the FST was determined to be 90.2 ± 3.6 and 127.6 ± 4.0 s, respectively (Figure 6b). The total immobility time of the control and CSDS C57BL/6J mice in the TST was 141.7 ± 4.8 and 190.2 ± 4.5 s, respectively (Figure 6c). The total immobility time of the control and CSDS C57BL/6J mice in the FST was 103.7 ± 4.9 and 157.5 ± 3.5 s, respectively (Figure 6d). These results demonstrated that the depression‐like behaviors had increased in the LPS‐treated CD‐1 mice and the C57BL/6J mice exposed to CSDS. The coefficients between the sleep time of LPS‐treated CD‐1 mice and that of C57BL/6J mice under CSDS were 92.4% and 93.1%, respectively, and their respective 95% limits of agreement were −615.5 to 703.1 and −531.0 to 575.8 s, respectively (Figure 6e–h). The total sleep time of the LPS‐treated CD‐1 mice was prolonged compared to that of the control group (Figure 6i,j), whereas the total sleep time of the C57BL/6J mice under CSDS was significantly shorter than that of the control group (Figure 6k,l).

### 3.6 Parameter consistency for all mice

**FIGURE 7:**
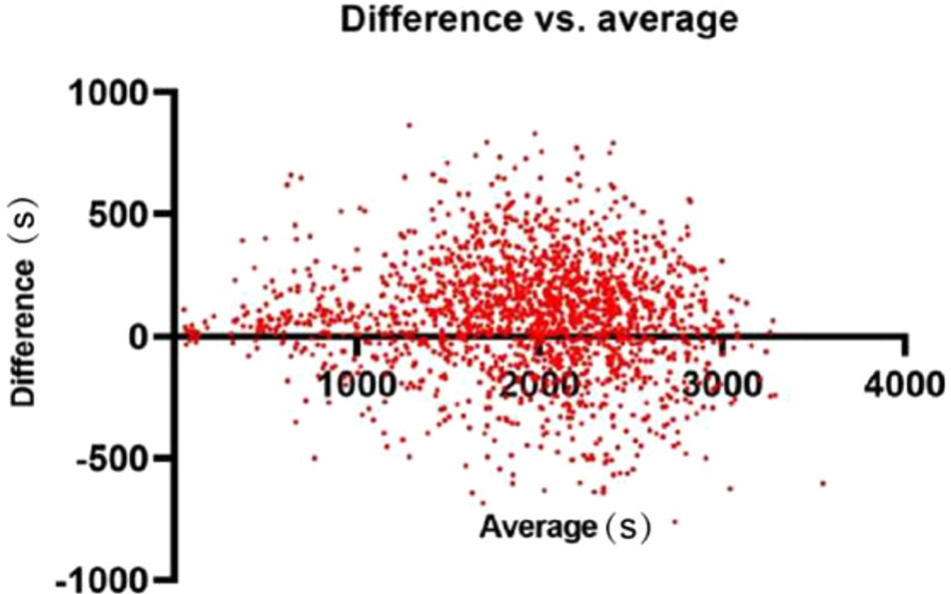
Summary of the consistency tests. Results of consistency tests for all the mice. The study involved a total of 13 groups, with 6 mice in each group. Each of the scatter points represent the difference between the results of sleep analysis obtained by electroencephalogram (EEG) and the video system in the same mouse within 1 h.

The coefficient between the sleep time detected by behavior‐based system and that determined by the EEG/EMG equipment was 93.64%. Based on the differences in sleep times under various conditions, the Bland–Altman 95% limits of agreement were determined to be −358.2 to 536.4 s (Figure 7).

## 4 DISCUSSION

In this study, by comparing video‐based immobility times with standard EEG/EMG sleep times, we preliminarily validated the feasibility of the video system for determining sleep–wake status in mice in various physiological, pharmacological, and pathological states. EEG/EMG, as a standard and classical sleep monitoring method, can obtain detailed sleep parameters and is widely used in the field of research (Jin et al., 2021; Monti, 2010; Takata et al., 2018). In contrast, the defects of the video system are more obvious, which can only obtain a preliminary sleep time and lose the rest of the sleep‐related characteristics. Compared to traditional EEG recording, the unique advantages of the video system are noninvasive, fast, and long duration observation. Therefore, video systems can be a suitable tool for initial monitoring of sleep time in certain situations.

In order to extensive validation, we combined the golden standard of sleep monitoring, namely, an EEG/EMG recording equipment, with video‐based system for simultaneously recording sleep behaviors. Inactivities for 35, 40, and 45 s in the recorded video were initially selected for defining sleep. The findings revealed that an inactivity of 40 s was the most appropriate parameter for defining sleep; therefore, this value was used in the subsequent experiments. Subsequent analysis revealed that the sleep times of mice detected by video‐based system were highly consistent with the sleep times obtained using the EEG/EMG equipment under different physiological conditions, and the coefficient between the methods was determined to be .94. In order to further explore the applicability of noninvasive sleep monitoring system, we included the CD‐1 mouse strain for simultaneous monitoring and found that the coefficient reached to .95.

Sleep and wakefulness are regulated by rhythms and endocytosis. The regulation of rhythm relies mainly on the regulation of neural circuits consisting of pro‐wake and pro‐sleep related brain regions in the brain. Endocytosis is mainly a number of substances that affect wakefulness and sleep, including adenosine and prostaglandin E, acetylcholine, 5‐HT, and dopamine. For example, diazepam inhibits sleep mainly by binding to gamma‐aminobutyric acid‐A (GABA‐A) receptors to inhibit the cerebral cortex and wakefulness‐related neurons (Kopp et al., 2003) and caffeine inhibits sleep primarily by blocking adenosine A2A receptors (Huang et al., 2014). Hence, we wanted to detect pharmacologically induced alterations in the sleep state. The findings revealed that the sleep times were reduced in C57BL/6J mice injected with relatively high doses of caffeine (10 and 15 mg/kg), whereas the sleep time of mice treated with a relatively low dose of caffeine (5 mg/kg) did not exhibit any marked difference with that of the control group. These findings indicated that noninvasive sleep monitoring system was capable of distinguishing between sleep and wakeful states and could detect the effect of different doses of caffeine on the sleep time. The results obtained in this study were consistent with the findings reported by Fisher et al. Different doses of diazepam were subsequently injected into the intraperitoneal cavity for determining the feasibility of application under drowsy conditions (Mckillop et al., 2021). The findings revealed that a relatively low dose (2.5 mg/kg) and a relatively high dose of diazepam (5 mg/kg) could prolong the sleep times of mice. Interestingly, comparison with the data obtained by EEG/EMG monitoring revealed that video‐based system did not overevaluate the sleep times of mice (refer to Section 3.3).

Exposure to predator odors can induce a stress response in animals and activate the hypothalamic–pituitary–adrenal (HPA) axis (Sotnikov et al., 2011). Previous studies have demonstrated that mice exposed to dirty cat litter display post‐traumatic stress disorder accompanied by excessive arousal (Sharma et al., 2018). We performed a similar experiment in this study and found that the stress induced by predator odor reduced the sleep time of mice (*t* = 11.29, *p* < .01), and video‐based system exhibited high sensitivity in detecting the reduction in sleep times following exposure to predator odor.

Animals under pathophysiological conditions usually have impaired motor functions and a longer duration of immobility. Previous studies have demonstrated that exposure to LPS or CSDS contributes to murine depression‐like behavior accompanied by the suppression of motor activity (Xu et al., 2020; Zhang et al., 2019). The LPS‐induced model of inflammation and the CSDS model are widely for studying the neurobiological mechanisms of depression. For further verification of the sleep/wake states in mice with depression, we therefore adopted the LPS‐induced model of inflammation and the CSDS model in this study (Jiang, Liu, et al., 2017; Jiang, Wang, et al., 2017) and assessed their sleep data. The models exhibited an increase in depression‐like behaviors, which were validated by TST and FST. Notably, we observed that the behavior‐based sleep monitoring system also worked in the mice with depression (refer to Section 3.5). Interestingly, we observed that the total sleep time in the LPS‐treated group was prolonged compared to that of the saline‐treated group (*t* = 4.49, *p* < .01), whereas the total sleep time of the CSDS group was shorter than that of the control mice in the sensory contact group (*t* = 8.30, *p* < .01). The differences could be attributed to the fact that exposure to pro‐inflammatory factors prolonged the sleep time in the LPS‐treated group (Tan et al., 2020), whereas the hyperactivity of the HPA axis reduced the sleep time in the CSDS group (Beins et al., 2021).

Altogether, we have supported the feasibility of behavioral‐based video sleep monitoring system to monitor sleep time through extensive validation under different physiological, pharmacological, and pathological conditions. Compared to traditional EEG recording systems, video‐based system has numerous advantages, which are discussed hereafter. First, noninvasive sleep monitoring system can be primarily used for the long‐term tracking of sleep times, whereas EEG/EMG equipment have a different range of application. Second, the use of EEG/EMG equipment does not allow for the complete screening of large samples of animals with insomnia or transgenic animals; however, undisturbed sleep data can be rapidly obtained using noninvasive sleep monitoring system. The most important feature of behavior‐based system is that it is completely noninvasive and therefore has a broader range of applications in pathological mouse models (Rancan et al., 2018) that cannot undergo the operative procedure necessary for implanting electrodes. Third, the video‐based system can be combined with other equipment by installing an infrared camera, which allows the tracking of corresponding behaviors associated with episodes of sleep/wakefulness. However, the device has some limitations for sleep monitoring based on mouse behaviors. First, it is unable to differentiate between NREM and REM sleep and provide information about sleep intensity. Second, the video‐based system does not have the same temporal resolution as EEG recording systems, which makes it impossible to analyze the physiological and behavioral changes during transitions between sleep and wakefulness. Third, Bland–Altman statistical method is inclusive, resulting in a high degree of consistency. Nonetheless, we must acknowledge that there is still a minor variation between video‐defined sleep time and EEG‐defined sleep time. Additionally, interventions that suppress sleep activity without causing a concomitant increase in wakefulness may give rise to false results. For instance, the activation of cholinergic neurons in the basal forebrain generates wakefulness waves from the cortex without obvious movement (Chen et al., 2016); therefore, the video‐based system might prove incapable in tracking the behaviors of mice under these circumstances for precisely detecting sleep/wakeful states.

## AUTHOR CONTRIBUTIONS

**Ya‐Tao Wang**: Conceptualization; data curation; formal analysis; methodology; validation; visualization; writing—original draft. **Yue‐Ming Zhang**: Conceptualization; data curation; formal analysis; methodology; validation; visualization; writing—original draft. **Xu Wu**: Conceptualization; data curation; formal analysis; methodology; software; validation; writing—original draft. **Chong‐Yang Ren**: Investigation; methodology. **Zhe‐Zhe Zhang**: Investigation; methodology. **Qi‐Gang Yang**: Conceptualization; methodology; project administration; resources; writing—review and editing. **Xue‐Yan Li**: Conceptualization; funding acquisition; methodology; project administration; resources; writing—review and editing. **Gui‐Hai Chen**: Conceptualization; funding acquisition; methodology; project administration; resources; writing—review and editing.

### PEER REVIEW

The peer review history for this article is available at https://publons.com/publon/10.1002/brb3.3311.

## Data Availability

The data used to support the findings of this study are available from the corresponding author upon request.
